# Mutagenic Study of Benzimidazole Derivatives with (+S9) and without (−S9) Metabolic Activation

**DOI:** 10.3390/ijms20184324

**Published:** 2019-09-04

**Authors:** Nurul Hafizan Azahar, Siti Soleha Ab dullah, Rozaini Abdullah, Norizan Ahmat, Abdah Md Akim, Hasiah Ab Hamid

**Affiliations:** 1 Department of Biomedical Sciences, Faculty of Medicine and Health Sciences, Universiti Putra Malaysia (UPM), Serdang 43400, Malaysia; 2 Department of Environmental and Occupational Health, Faculty of Medicine and Health Sciences, Universiti Putra Malaysia (UPM), Serdang 43400, Malaysia; 3 School of Chemistry and Environment, Faculty of Applied Sciences, Universiti Teknologi MARA (UiTM), Shah Alam 40450, Malaysia

**Keywords:** benzimidazole, Ames test, structure–activity relationship (SAR)

## Abstract

Benzimidazole derivatives have a diverse range of biological activities, including antiulcer, antihypertensive, antiviral, antifungal, anti-inflammatory, and anticancer. Despite these activities, previous studies have revealed that some of the derivatives can induce mutations. This study aimed to screen for potential mutagenic activities of novel benzimidazole derivatives 1–4 using the Ames test and to study their structure–activity relationship (SAR). An Ames test was carried out on two strains of *Salmonella typhimurium* (TA98 and TA100) in the absence and presence of metabolic activation. Genetic analysis was performed prior to the Ames test to determine the genotypes of the bacterial tester strains. Both bacterial strains showed dependency on histidine with the presence of *rfa* mutation, *uvr*B deletion, and plasmid pKM101. Further, all derivatives tested showed no mutagenic activity in the absence of metabolic activation in both tester strains. However, in the presence of metabolic activation, compound 1 appeared to induce mutation at 2.5 µg/plate when tested against the TA98 strain. These results suggest that the absence of the -OH group at the *ortho*-position over the phenyl ring might be the cause of increased mutagenic activity in compound 1. Additionally, the presence of mutagenic activity in compound 1 when it was metabolically activated indicates that this compound is a promutagen.

## 1. Introduction

Mutations can be defined as changes in the nucleotide sequence of DNA which can lead to various genetic disorders and severe diseases. There are two types of gene mutations: point and frameshift mutations. Point mutations arise when there are changes in a single nucleotide of the DNA sequence, such as transition and transversion. On the other hand, frameshift mutations occur when several nucleotides are inserted or deleted in the DNA sequence. Any substances that can cause irreversible and heritable changes to the DNA of an organism are known as mutagens, for instance, chemicals and radiation. Mutations can also occur spontaneously due to errors in DNA replication which remain unfixed throughout cell division. Mutations that occur in germ cells can be passed on to future generations, while somatic mutations give rise to many genetic variations and pathological conditions, including cancer [1,2,3,4].

The Ames test is an inexpensive, time-efficient, and convenient bacterial reversion assay that employs bacteria, either *Salmonella typhimurium* or *Escherichia coli*, to assess the mutagenic potential of new chemical substances. Identification of mutagens has become a major concern in research, especially when it is associated with drug discovery and development [3]. A positive result on the Ames test indicates the potential induction of mutations by the compound of interest. This result may halt the development of potential therapeutic agents into drugs [5,6].

**Figure 1:**
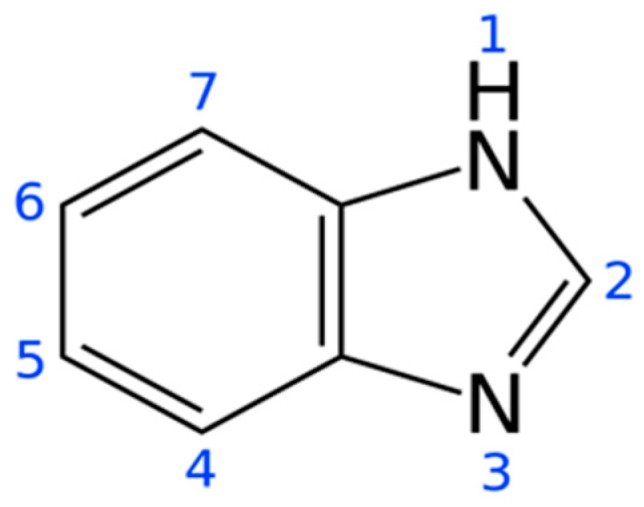
Structure of benzimidazole.

Benzimidazole is a heterocyclic aromatic organic compound which consists of a benzene ring and imidazole ring (Figure 1). It has a diverse range of biological activities, including antiviral, anthelminthic, antimicrobial, analgesic, anti-inflammatory, antidiabetic, antihypertensive, and anticancer [7,8,9]. Due to its remarkable potential on various disease models, many modifications have been carried out around its backbone in order to improve its biological activities, thus resulting in many of its derivatives [8,10]. Some of the series of newly synthesized benzimidazole hydrazone derivatives have demonstrated significant antidiabetic activity [11]. A study by Refaat (2010) revealed the potential antitumor activity of a new series of 2-substituted benzimidazole derivatives against HepG2, MCF-7, and HCT-116 cancer cell lines [12]. Likewise, Wu et al. (2016) reported strong antiproliferative activities of a series of benzimidazole-2-substituted phenyl or pyridine propyl ketene derivatives against HepG2, MCF-7, and HCT-116 cells [13].

Despite their therapeutic activities, previous studies had demonstrated that some of the benzimidazole derivatives can induce mutations [14,15]. Even though there are only a few studies that show the mutagenic potential of benzimidazole derivatives, it was still crucial to carry out a mutagenicity assessment of our compound of interest. The presence of the mutagenic activity in any therapeutic agent may affect the commencement of clinical trials [3].

**Figure 2:**
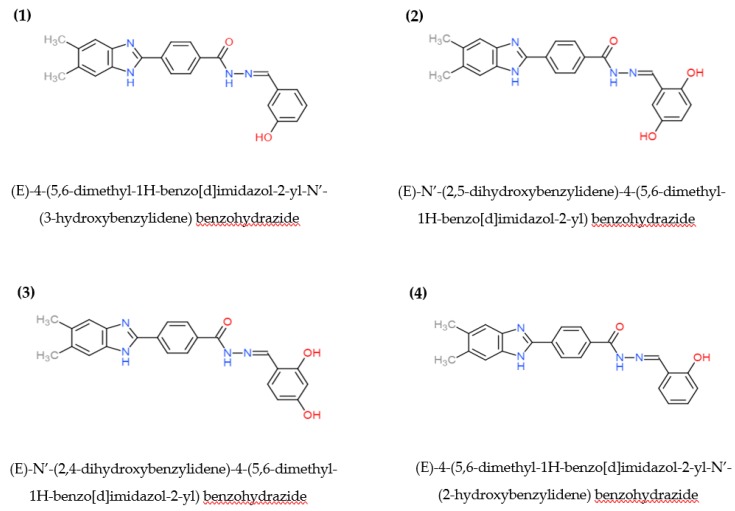
Chemical structure of benzimidazole derivatives.

Hence, this study was carried out in order to screen for potential mutagenic activities of novel benzimidazole derivatives 1–4 (Figure 2) using the Ames test, as well as to study their structure–activity relationship (SAR).

## 2. Results

### 2.1. Genetic Analysis

The results of genetic analysis, as presented in Table 1, were in agreement with the guidelines provided by Maron and Ames [16] and Mortelmans and Zeiger [1]. Both bacterial tester strains (TA98 and TA100) showed dependency on histidine with the presence of *rfa* mutation, *uvr*B deletion, and plasmid pKM101.

### 2.2. Mutagenicity Assay

The numbers of revertant colonies (expressed as mean ± SEM) of TA98 and TA100 strains of *S. typhimurium* after treatment with different doses of benzimidazole derivatives 1–4 are summarized in Table 2 and Table 3, respectively, in the absence and presence of metabolic activation system. The mutagenic indexes (MIs) were calculated and the values are shown in parentheses.

**Figure 3:**
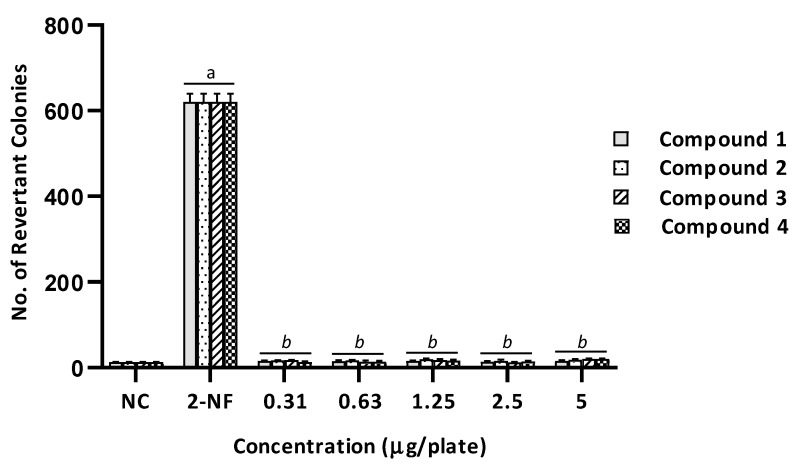
The number of revertant colonies of TA98 strain of *Salmonella typhimurium* treated with different concentration of benzimidazole derivatives (1–4) in the absence of metabolic activation system (−S9). NC, Negative control (1% of DMSO in PBS); 2-NF (2-Nitrofluorene), Positive control (2.5 µg/plate). Values were expressed as mean ± SEM of three independent experiments. a, significantly different (*p* < 0.05) from the NC group; *b*, significantly different (*p* < 0.05) from the positive control group (2-NF).

**Figure 4:**
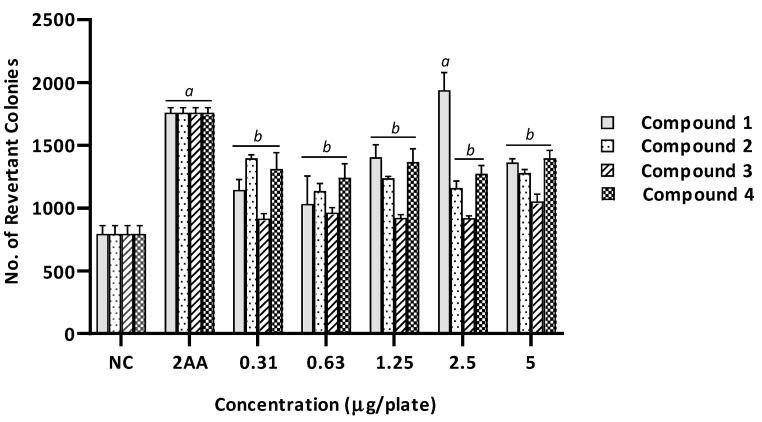
The number of revertant colonies of TA98 strain of *Salmonella typhimurium* treated with different concentration of benzimidazole derivatives (1–4) in the presence of metabolic activation system (+S9). NC, Negative control (1% of DMSO in PBS); 2AA (2-aminoanthracene), Positive control (5 µg/plate). Values were expressed as mean ± SEM of three independent experiments. *a*, significantly different (*p* < 0.05) from the NC group; *b*, significantly different (*p* < 0.05) from the positive control group (2AA).

Based on the results, no mutagenicity was observed in the absence of metabolic activation in all benzimidazole derivatives at any dose tested on the TA98 strain. There was no significant difference in the number of revertant colonies between the treated groups and the negative control group (*p* > 0.05), as shown in Figure 3. On the contrary, compound 1 showed mutagenic potential in the presence of metabolic activation at 2.5 µg/plate with the MI of 2.2 (Table 2). There was a significant increase in the number of revertant colonies compared with the negative control group (*p* < 0.05), as shown in Figure 4.

**Figure 5:**
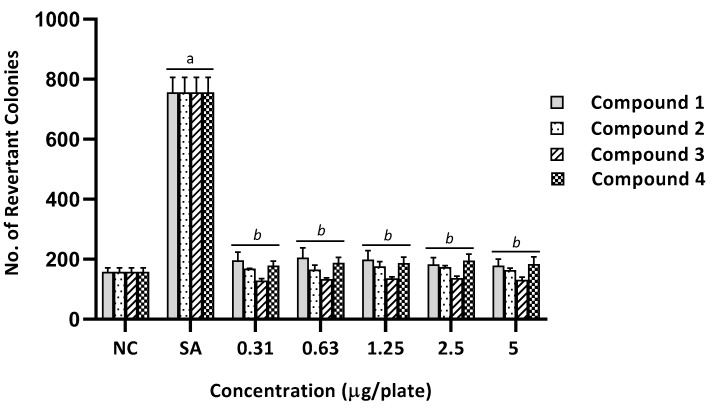
The number of revertant colonies of TA100 strain of *Salmonella typhimurium* treated with different concentration of benzimidazole derivatives (1–4) in the absence of metabolic activation system (−S9). NC, Negative control (1% of DMSO in PBS); SA (sodium azide), Positive control (5 µg/plate). Values were expressed as mean ± SEM of three independent experiments. a, significantly different (*p* < 0.05) from the NC group; *b*, significantly different (*p* < 0.05) from the positive control group (SA).

**Figure 6:**
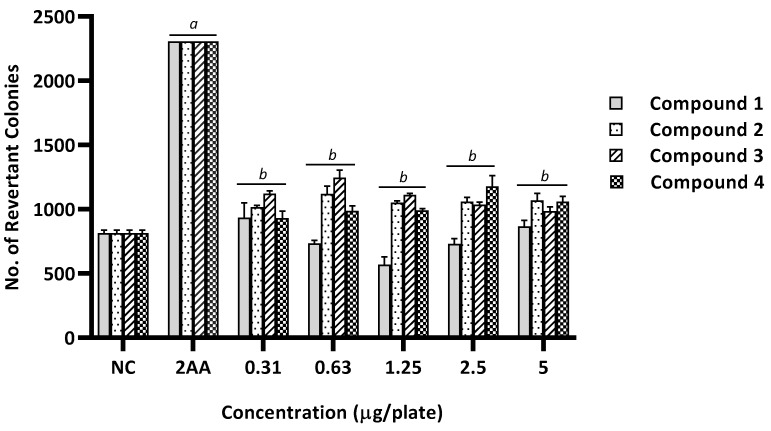
The number of revertant colonies of TA100 strain of *Salmonella typhimurium* treated with different concentration of benzimidazole derivatives (1–4) in the presence of metabolic activation system (+S9). NC, Negative control (1% of DMSO in PBS); 2AA (2-aminoanthracene), Positive control (5 µg/plate). Values were expressed as mean ± SEM of three independent experiments. *a*, significantly different (*p* < 0.05) from the NC group; *b*, significantly different (*p* < 0.05) from the positive control group (2AA).

In the TA100 strain, all derivatives were not mutagenic at any of the tested doses, with and without metabolic activation, as presented in Table 3 and Figure 5 and Figure 6. None of the derivatives showed an MI ≥ 2, suggesting the absence of induced mutagenic activity following treatment.

## 3. Discussion

The potential mutagenic activity of benzimidazole derivatives was evaluated using the Ames test in the absence and presence of metabolic activation. The Ames test, which was introduced in 1973 by Bruce N. Ames, is a short-term bacterial reversion assay that employs specially constructed mutants of *S. typhimurium* to ascertain the mutagenic potential of new chemical substances. This assay is quicker and less expensive than traditional animal testing and tissue culture [17]. Using this assay, two customized tester strains of *S. typhimurium* were used (TA98 and TA100) to check for frameshift and base-pair substitution mutations, respectively. These bacterial tester strains contain different mutations in their histidine operon, which make them unable to produce histidine to survive. In the presence of mutagenic chemicals, these mutated bacterial strains revert to their original state, restoring their ability to produce histidine. As a result, the growth of bacterial colonies can be observed on a minimal agar plate containing a small amount of histidine [1,16,17,18,19].

In the presence of metabolic activation, the S9 fraction was added together with the test compound and bacteria. The S9 fraction consists of liver homogenate from rats treated with the Aroclor-1254 to increase the level of hepatic metabolizing enzymes. Addition of S9 metabolic activation system in the experiment enables detection of potential promutagens. Some chemicals display mutagenicity only after metabolism by phase 1 drug-metabolizing enzymes, which mainly belong to the cytochrome P450 family. Since bacteria do not possess this metabolic capability, the S9-based metabolic activation system needs to be introduced in this assay [1,3].

As presented in Table 2 and Table 3, there was no mutagenic activity observed in all benzimidazole derivatives (1–4), at any tested dose, in both standard tester strains of *S. typhimurium* (TA98 and TA100) in the absence of the S9 metabolic activation system. None of the tester strains showed an MI ≥ 2 or any dose–response effect, suggesting the absence of potential mutagenic activity [6,18,20]. Moreover, the numbers of revertant colonies were comparable between all treated groups and the negative control group, as illustrated in Figure 3 and Figure 5.

Likewise, benzimidazole derivatives were found to be devoid of mutagenic activity when tested in the presence of the S9 metabolic activation system using the TA100 strain (Table 3 and Figure 6). There was no significant increase in the number of revertant colonies in all treated groups in comparison to the negative control group (*p* > 0.05). Furthermore, none of the MIs reached 2, suggesting that the benzimidazole derivatives did not induce base-pair substitution mutations. In contrast, one of the benzimidazole derivatives (compound 1) appeared to induce mutagenicity in the presence of the S9 metabolic activation system, at the concentration of 2.5 µg/plate with an MI of 2.2, when tested against the TA98 strain (Table 2 and Figure 4). The number of revertant colonies increased by more than twofold in comparison with the negative control group, which was comparable to the number of revertant colonies induced by the positive control (2-aminoanthracene). This result indicated a potential induction of frameshift mutation by compound 1 at the concentration of 2.5 µg/plate when the metabolic activation was present.

Referring to the chemical structure shown in Figure 2, all derivatives shared a similar structure except for the number and position of the hydroxyl groups (-OH) attached to the phenyl ring. One hydroxyl group was attached at the *ortho*- and *meta*-positions of the phenyl ring for compounds 4 and 1, respectively, while two hydroxyl groups were attached at the *ortho*–*meta*- and *ortho*–*para*-positions over the phenyl ring for compounds 2 and 3, respectively [11]. In the absence of metabolic activation, all benzimidazole derivatives showed no significant difference in mutagenic activity (MI < 2) despite their differences in the number and position of the hydroxyl groups.

Conversely, with the presence of metabolic activation, one of the derivatives (compound 1) was found to induce mutation only at 2.5 µg/plate in the TA98 strain. When compared to the structure of other derivatives, *ortho*-OH was not present on the phenyl ring of compound 1. This result suggested that *ortho*-OH substitution may possibly affect the mutagenic activity of benzimidazole derivatives.

In summary, all benzimidazole derivatives (1–4) exhibited no mutagenic activity in both tester strains (TA98 and TA100) in the absence of metabolic activation, despite the dissimilarity in the number and position of the -OH groups. However, in the presence of metabolic activation, only compound 1 appeared to induce mutation at 2.5 µg/plate when tested against the TA98 strain. The lack of the -OH group at the *ortho*-position over the phenyl ring might be the cause of the increased mutagenic activity in compound 1. The presence of mutagenic activity in compound 1 when it was metabolically activated suggests that this compound is a promutagen.

However, a limitation of this study is that we used *S. typhimurium*, a prokaryote, to screen for potential mutagenic activities. A prokaryote is a unicellular organism; therefore, it is not a perfect model to study mutagenicity in humans. The results obtained from this assay might be different if higher organisms were used. Nevertheless, this assay provides valuable preliminary data on the mutagenic potency of the tested chemicals.

## 4. Materials and Methods

### 4.1. Chemicals

Novel benzimidazole derivatives were synthesized at Universiti Teknologi MARA (UiTM), Shah Alam, Malaysia. D-biotin, L-histidine, nicotinamide adenine dinucleotide phosphate (NADP), 2-nitrofluorene (2-NF), 2-aminoanthracene (2AA), and S9 from pooled livers of Sprague-Dawley male rats were purchased from Sigma-Aldrich (St. Louis, MO, USA). Sodium azide was obtained from Merck (Darmstadt, Germany). Nutrient broth no. 2, nutrient agar, dextrose, and agar bacteriological (agar no. 1) were purchased from Oxoid Ltd (Basingstoke, Hampshire, England).

### 4.2. Bacterial Strains

TA98 and TA100 strains of *S. typhimurium* were purchased from the American Type Culture Collection (ATCC) (USA). An inoculum (12 µL) from a frozen stock of bacteria was added to 12 mL of nutrient broth (Oxoid nutrient broth no. 2) before being incubated at 37 °C in a shaking water bath at 100 rpm for 12–14 h.

### 4.3. Genetic Analysis

Genetic analysis was carried out as reported by Maron and Ames (1983) in order to confirm the genotypes of the bacterial strains used [16]. This analysis included the dependency of the bacterial tester strains on histidine or biotin, as well as the presence of *rfa* mutation, *uvr*B deletion, and plasmid pKM101.

### 4.4. S9 Metabolic Activation System

The S9 fraction from pooled livers of Sprague-Dawley male rats was purchased from Sigma-Aldrich, USA. The metabolic activation system (4%) was prepared by adding sterilized distilled water, 0.2 M phosphate buffer (pH 7.4), 0.1 M nicotinamide adenine dinucleotide phosphate sodium (NADP), 1 M glucose-6-phosphate, MgCl_2_-KCl salt solution, and S9 fraction in a sequential manner. The preparation was carried out on ice in a dark environment.

### 4.5. Mutagenicity Assay

The mutagenicity assay was performed as described by Maron and Ames (1983) with a slight modification [16]. All derivatives of benzimidazole—(E)-4-(5,6-dimethyl-1H-benzo[d]imidazol-2-yl)-N’-(3-hydroxybenzylidene) benzohydrazide (1), (E)-N’-(2,5-dihydroxybenzylidene)-4-(5,6-dimethyl-1H-benzo[d]imidazol-2-yl) benzohydrazide (2), (E)-N’-(2,4-dihydroxybenzylidene)-4-(5,6-dimethyl-1H-benzo[d]imidazol-2-yl) benzohydrazide (3), and (E)-4-(5,6-dimethyl-1H-benzo[d]imidazol-2-yl)-N’-(2-hydroxybenzylidene) benzohydrazide (4)—were preincubated with TA98 and TA100 bacterial strains for 20 min, with and without metabolic activation. All derivatives were tested at five different concentrations: 5, 2.5, 1.25, 0.63, and 0.31 µg/plate.

Various concentrations of the tested benzimidazole derivatives were added to a mixture of 500 µL of 0.1 M phosphate buffer, pH 7.4 (without metabolic activation), or 500 µL of the S9 mix (with metabolic activation) and 100 µL of bacterial culture. Then, the mixture was incubated at 37 °C for 20 min. After incubation, 2 mL of melted top agar was added to the mixture and poured immediately on a GM agar plate. All plates were incubated at 37 °C for another 48 h before the revertant colonies were counted. The experiment was carried out in triplicates.

The positive controls that were used in the experiment without metabolic activation were 2.5 µg/plate of 2-nitrofluorene (TA98) and 5 µg/plate of sodium azide (TA100). As for the experiment with the presence of metabolic activation, 5 µg/plate of 2-aminoanthracene was used for both strains. The mixture of 1% DMSO in phosphate buffered saline (PBS) served as a negative control in the experiments.

### 4.6. Data Analysis

All data were analyzed by using two-way ANOVA, followed by a post hoc Tukey’s test. The MI was calculated by dividing the average number of revertant colonies per plate (from test plates) with the average number of revertant colonies per plate (from negative control plates). The result was considered to be mutagenic when the MI was equal to or greater than 2 (MI ≥ 2) in at least one of the tested doses [6,18,20]. The SAR was analyzed based on the results obtained.

$$\mathrm{MI}=\frac{\mathrm{Average}\;\mathrm{number}\;\mathrm{of}\;\mathrm{revertant}\;\mathrm{colonies}\;\mathrm{in}\;\mathrm{test}\;\mathrm{plates}}{\mathrm{Average}\;\mathrm{number}\;\mathrm{of}\;\mathrm{revertant}\;\mathrm{colonies}\;\mathrm{in}\;\mathrm{negative}\;\mathrm{control}\;\mathrm{plates}}$$

## Figures and Tables

**Table 1:** Genetic analysis of TA98 and TA100 strains of *Salmonella typhimurium*.

Genetic Properties	Presence of Bacterial Growth on Plate	
	TA98	TA100
1. Histidine dependence	x	x
2. Biotin dependence	/	/
3. Presence of *rfa* mutation	x	x
4. Presence of *uvr*B deletion	x	x
5. Presence of plasmid pKM101	/	/

(x) shows absence of bacterial growth, whereas (/) shows presence of bacterial growth on plate.

**Table 2:** The number of revertant colonies of TA98 strain of *Salmonella typhimurium* in the absence (−S9) and presence (+S9) of metabolic activation system.

Compounds	Concentration (µg/plate)													
	NC		PC		0.31		0.63		1.25		2.5		5	
	−S9	+S9	−S9	+S9	−S9	+S9	−S9	+S9	−S9	+S9	−S9	+S9	−S9	+S9	
**1**	14 ± 2 (1.0)	876 ± 83 (1.0)	644 ± 6 (46) ^a^	1787 ± 82 (2.0) ^a^	15 ± 2 (1.1)	1144 ± 85 (1.3)	15 ± 3 (1.0)	1033 ± 223 (1.2)	15 ± 2 (1.1)	1405 ± 99 (1.6)	14 ± 3 (1.0)	1939 ± 142 (2.2) ^a^	15 ± 3 (1.1)	1363 ± 31 (1.6)	
**2**	12 ± 2 (1.0)	816 ± 79 (1.0)	561 ± 7 (45.5) ^a^	1787 ± 82 (2.2) ^a^	17 ± 1 (1.4)	1396 ± 28 (1.7)	17 ± 2 (1.4)	1137 ± 60 (1.4)	20 ± 2 (1.6)	1239 ± 13 (1.5)	15 ± 3 (1.2)	1160 ± 56 (1.4)	18 ± 3 (1.5)	1280 ± 28 (1.6)	
**3**	13 ± 3 (1.0)	675 ± 18 (1.0)	631 ± 12 (48.5) ^a^	1680 ± 106 (2.5) ^a^	17.± 1 (1.3)	916 ± 39 (1.4)	14 ± 3 (1.1)	965 ± 39 (1.4)	18 ± 2 (1.4)	923 ± 25 (1.4)	12 ± 2 (1.0)	920 ± 19 (1.4)	20 ± 2 (1.5)	1053 ± 57 (1.6)	
**4**	14 ± 2 (1.0)	816 ± 79 (1.0)	644 ± 6 (46) ^a^	1787 ± 82 (2.2) ^a^	13 ± 2 (0.9)	1311 ± 132 (1.6)	14 ± 3 (1.0)	1243 ± 111 (1.5)	16 ± 2 (1.2)	1368 ± 104 (1.7)	14 ± 2 (1.0)	1273 ± 66 (1.6)	20 ± 2 (1.4)	1396 ± 64 (1.7)	

NC, Negative control (1% of DMSO in PBS); PC, Positive controls for TA98/−S9 (2.5 µg/plate of 2-nitrofluorene) and TA98/+S9 (5 µg/plate of 2-aminoanthracene); values in parentheses indicate the mutagenic index (MI). All values were expressed as mean ± SEM of three independent experiments which carried out in duplicate. ^a^, significantly different (*p* < 0.05) from the NC group.

**Table 3:** The number of revertant colonies of TA100 strain of *Salmonella typhimurium* in the absence (−S9) and presence (+S9) of metabolic activation system.

Compounds	Concentration (µg/plate)													
	NC		PC		0.31		0.63		1.25		2.5		5	
	−S9	+S9	−S9	+S9	−S9	+S9	−S9	+S9	−S9	+S9	−S9	+S9	−S9	+S9
**1**	172 ± 23 (1.0)	821 ± 32 (1.0)	676 ± 5 (3.9) ^a^	2307 ± 202 (2.8) ^a^	196 ± 28 (1.1)	934 ± 116 (1.1)	205 ± 33 (1.2)	735 ± 23 (0.9)	199 ± 30 (1.2)	570 ± 59 (0.7)	183 ± 23 (1.1)	731 ± 40 (0.9)	178 ± 22 (1.0)	867 ± 47 (1.1)
**2**	169 ± 4 (1.0)	768 ± 94 (1.0)	788 ± 28 (4.7) ^a^	2307 ± 202 (3.0) ^a^	168 ± 2 (0.1)	1017 ± 13 (1.3)	166 ± 15 (1.0)	1120 ± 61 (1.5)	176 ± 16 (1.1)	1051 ± 15 (1.4)	174 ± 5 (1.0)	1060 ± 32 (1.4)	164 ± 6 (1.0)	1069 ± 54 (1.4)
**3**	119 ± 5 (1.0)	897 ± 5 (1.0)	885 ± 27 (7.5) ^a^	2307 ± 202 (2.6) ^a^	129 ± 7 (1.1)	1121 ± 21 (1.2)	133 ± 4 (1.1)	1246 ± 58 (1.4)	136 ± 6 (1.2)	1112 ± 12 (1.2)	137 ± 6 (1.2)	1036 ± 19 (1.2)	131 ± 10 (1.1)	985 ± 34 (1.1)
**4**	172 ± 23 (1.0)	768 ± 94 (1.0)	676 ± 5 (3.9) ^a^	2307 ± 202 (3.0) ^a^	178 ± 15 (1.0)	931 ± 55 (1.2)	188 ± 19 (1.1)	987 ± 40 (1.3)	187 ± 20 (1.1)	991 ± 15 (1.3)	195 ± 23 (1.1)	1179 ± 83 (1.5)	183 ± 25 (1.1)	1060 ± 40 (1.4)

NC, Negative control (1% of DMSO in PBS); PC, Positive controls for TA100/−S9 (5 µg/plate of sodium azide) and TA100/+S9 (5 µg/plate of 2-aminoanthracene); values in parentheses indicate the mutagenic index (MI). All values were expressed as mean ± SEM of three independent experiments which carried out in duplicate. ^a^, significantly different (*p* < 0.05) from the NC group.

## References

[1] Mortelmans K., Zeiger E. (2000). The Ames *Salmonella*/microsome mutagenicity assay. Mutat. Res..

[2] Loeb K.R., Loeb L.A. (2000). Significance of multiple mutations in cancer. Carcinogenesis.

[3] Słoczyńska K., Powroźnik B., Pękala E., Waszkielewicz A.M. (2014). Antimutagenic compounds and their possible mechanisms of action. J. Appl. Genet..

[4] Verheyen G.R., Deun K.V., Miert S.V. (2017). Testing the mutagenicity potential of chemicals. J. Genet. Genome Res..

[5] McCarren P., Springer C., Whitehead L. (2011). An investigation into pharmaceutically relevant mutagenicity data and the influence on Ames predictive potential. J. Cheminformatics.

[6] Anjum R., Krakat N., Reza M.T., Klocke M. (2014). Assessment of mutagenic potential of pyrolysis biochars by Ames Salmonella/mammalian-microsomal mutagenicity test. Ecotoxicol. Environ. Saf..

[7] Bansal Y., Silakari O. (2012). The therapeutic journey of benzimidazoles: A review. Bioorganic Med. Chem..

[8] El Rashedy A.A., Aboul-Enein H.Y. (2013). Benzimidazole derivatives as potential anticancer agents. Mini Rev. Med. Chem..

[9] Pullagura M.K.P., Kanvinde A., Raja S. (2016). Potent Biological Agent Benzimidazole-A Review. Int. J. Pharm. Pharm. Sci..

[10] Rao G.E., Babu P.S., Koushik O.S., Sharmila R., Vijayabharathi M., Maruthikumar S., Pavankumar P. (2016). A review on chemistry of benzimidazole nucleus and its biological significance. Int. J. Pharm. Chem. Biol. Sci..

[11] Zawawi N.K.N.A., Taha M., Ahmat N., Wadood A., Ismail N.H., Rahim F., Abdullah N. (2016). Benzimidazole derivatives as new α-glucosidase inhibitors and in silico studies. Bioorganic Chem..

[12] Refaat H.M. (2010). Synthesis and anticancer activity of some novel 2-substituted benzimidazole derivatives. Eur. J. Med. Chem..

[13] Wu L.T., Jiang Z., Shen J.J., Yi H., Zhan Y.C., Sha M.Q., Li Z.R. (2016). Design, synthesis and biological evaluation of novel benzimidazole-2-substituted phenyl or pyridine propyl ketene derivatives as antitumour agents. Eur. J. Med. Chem..

[14] Gümüş F., Demirci A.B., Özden T., Eroğlu H., Diril N. (2003). Synthesis, characterization and mutagenicity of new cis-[Pt (2-substituted-benzimidazole) 2Cl2] complexes. Pharmazie.

[15] Alanyalı F.S., Arıcı M., Artagan Ö., Işıkdağ İ., Özkay Y. (2010). Mutagenicity of Bisbenzimidazole Derivatives. Z. Naturforsch C..

[16] Maron D.M., Ames B.N. (1983). Revised methods for the Salmonella mutagenicity test. Mutat. Res..

[17] Creager A.N.H., Boudia S., Jas N., Boudia S., Jas N. (2014). The Political Life of Mutagens: A History of the Ames Test. Powerless Science? Science and Politics in a Toxic World.

[18] Santos J.L., Bosquesi P.L., Almeida A.E., Chin C.M., Varanda E.A. (2011). Mutagenic and genotoxic effect of hydroxyurea. Int. J. Biomed. Sci.: IJBS.

[19] Vijayan V., Pathak U., Meshram G.P. (2014). Mutagenicity and antimutagenicity studies of DRDE-07 and its analogs against sulfur mustard in the in vitro Ames Salmonella/microsome assay. Mutat. Res..

[20] Carneiro C.C., Véras J.H., Góes B.R., Pérez C.N., Chen-Chen L. (2018). Mutagenicity and antimutagenicity of Salacia crassifolia (mart. Ex. Schult.) G. Don. evaluated by Ames test. Braz. J. Biol..

